# A simplicial complex-based approach to unmixing tumor progression data

**DOI:** 10.1186/s12859-015-0694-x

**Published:** 2015-08-12

**Authors:** Theodore Roman, Amir Nayyeri, Brittany Terese Fasy, Russell Schwartz

**Affiliations:** 1 Computatational Biology Department, Carnegie Mellon University, 5000 Forbes Ave., Pittsburgh, USA; 2 Computer Science Department, Carnegie Mellon University, 5000 Forbes Ave., Pittsburgh, USA; 3 Department of Computer Science, Tulane University, 6834 St. Charles St., New Orleans, USA; 4 Department of Biological Sciences, Carnegie Mellon University, 5000 Forbes Ave., Pittsburgh, USA

**Keywords:** Cancer, Tumor phylogeny, Mixture modeling, Computational geometry, Genomics

## Abstract

**Background:**

Tumorigenesis is an evolutionary process by which tumor cells acquire mutations through successive diversification and differentiation. There is much interest in reconstructing this process of evolution due to its relevance to identifying drivers of mutation and predicting future prognosis and drug response. Efforts are challenged by high tumor heterogeneity, though, both within and among patients. In prior work, we showed that this heterogeneity could be turned into an advantage by computationally reconstructing models of cell populations mixed to different degrees in distinct tumors. Such mixed membership model approaches, however, are still limited in their ability to dissect more than a few well-conserved cell populations across a tumor data set.

**Results:**

We present a method to improve on current mixed membership model approaches by better accounting for conserved progression pathways between subsets of cancers, which imply a structure to the data that has not previously been exploited. We extend our prior methods, which use an interpretation of the mixture problem as that of reconstructing simple geometric objects called simplices, to instead search for structured unions of simplices called simplicial complexes that one would expect to emerge from mixture processes describing branches along an evolutionary tree. We further improve on the prior work with a novel objective function to better identify mixtures corresponding to parsimonious evolutionary tree models. We demonstrate that this approach improves on our ability to accurately resolve mixtures on simulated data sets and demonstrate its practical applicability on a large RNASeq tumor data set.

**Conclusions:**

Better exploiting the expected geometric structure for mixed membership models produced from common evolutionary trees allows us to quickly and accurately reconstruct models of cell populations sampled from those trees. In the process, we hope to develop a better understanding of tumor evolution as well as other biological problems that involve interpreting genomic data gathered from heterogeneous populations of cells.

**Electronic supplementary material:**

The online version of this article (doi:10.1186/s12859-015-0694-x) contains supplementary material, which is available to authorized users.

## Background

Cancer progression is an evolutionary process of successive genetic diversification and selection for mutations promoting tumor growth. While specific sequences of mutations are idiosyncratic to each tumor, functionally similar clusters of mutations are found across many patients in specific genetic pathways that are crucial to promoting tumor growth and invasion and disabling normal checks on cancer development [1]. The recognition that distinct tumors frequently exhibit similar progression pathways led to the idea of cancer phylogenetics (oncogenetics): the use of algorithms for evolutionary tree-building to reconstruct common pathways of evolution in tumors [2]. These methods originally modeled distinct tumors as “species,” using heterogeneity between tumors to derive phylogenetic trees providing an inference of common progresion pathways among patients. An alternative approach to tumor phylogenetics arose from the observation that as tumors evolve, they generate heterogeneity between cell populations within single tumors. This cell-to-cell variability can also be used to infer pathways of tumor evolution, in this case via phylogenetic trees linking cell populations in single tumors [3, 4].

Both strategies have inspired considerable subsequent study, but each also brings some significant limitations. Approaches drawing phylogenetic inferences from intertumor heterogeneity are generally limited by the fact that the bulk genomic data used by such approaches conflate all cells within a tumor (or subregion of a tumor [5–7]) into a single mixed profile, hiding variability cell-to-cell that includes many important clues about tumor progression. Studies building phylogenies of single tumors from intratumor heterogeneity can produce much finer reconstruction of cell-to-cell variation, but generally with more limited profiles of the individual states in an evolutionary tree. Much of the work on cellular tumor phylogenetics to date has relied on fluourescence in situ hybridization (FISH) [3, 4, 8, 9], a technology that makes it practical to examine thousands of single cells but to observe only a few pre-chosen markers per cell. The more recent introduction of single-cell sequencing for cancer studies [10] has made it possible in principle to observe and to apply whole-genome genetic variation data on single-cells for phylogenetic inference, but it, too, is limited so far by poor data quality and coverage [11] and a high cost that has made it necessary for each such study to profile only small numbers of tumors [10] or small numbers of cells per tumor [12–14].

In prior work, we proposed that one could in principle combine the advantages of both intertumor and intratumor phylogenetics through a class of computational methods called “mixed membership modeling” [15] (also known as unmixing or deconvolution) that can computationally infer patterns of intratumor variability from bulk genomic tumor data [16]. (We note that such models are sometimes called “mixture models” in this literature, although that term more properly refers to models in which each data point has a single potentially uncertain class.) Such mixed membership models provide a way to reconstruct whole-genome profiles of major cell populations in tumors, constructing models of major progression steps both within and across tumor populations simultaneously solely from intertumor genomic data. Similar mixed membership model approaches were initially used in cancer research to distinguish confounding influences of genetically healthy stromal cells in interpreting tumor genomic assays [17–19] and later extended to resolve multiple clonal states in tumor samples [20] independently of their use for tumor phylogenetics. Our specific use of mixture modeling for tumor phylogenetics relied on a geometric intepretation of unmixing as the problem of finding bounding simplices around point clouds of tumor genomic data [21], an approach to mixed membership modeling also known as archetype analysis [22].

This mixed membership approach to tumor phylogenetics has itself inspired extensive recent work on improved reconstruction of cell populations and progression pathways and application to wide varieties of bulk genomic data types. This work has included further development of the geometric approaches [23] as well as numerous other strategies for deconvolution of tumor genomic data. Earlier versions of this approach focused on the related problem of purity estimation, essentially assuming each tumor is a mixture of normal cells with a single class of tumor cell. Oesper et al. [24] provides a recent example working from DNA sequence data. More closely related to our work has been a variety of techniques for reconstructing more detailed subclonal architectures within tumors. For example, Zare et al. [25] provided a technique to derive tumor phylogenies (also called oncogenetic trees) from next generation sequencing (NGS) reads of regional sections of single tumors. Ha et al. [26] developed an approach incorporating loss of heterozygosity (LOH) events into a probabilistic framework for interpreting mixtures of sequence data. Li et al. [27] likewise developed a mixed membership approach to integrate subclone and purity estimation from NGS data and LOH data. Roth et al. [28] developed Bayesian models specificaly tuned for deep (> 100X) sequencing. The work of Qiao and others [29] focused on using variant allele frequencies to generate oncogenetic trees, but using the relative prevalences of subclones as an input. Such methods have been applied to a wide variety of genomic data types, including RNA expression [16, 30], copy number variant (CNV) [23, 24, 26, 28, 31], and single nucleotide variant (SNV) [20, 28, 30, 32–34] data. As bulk tumor sequencing data has become available on large scales (c.f., [35]), descendants of these mixture approaches have proven particularly popular as a way of deconvolving reads from bulk NGS data, including the exemplar approaches mentioned above and a variety of related methods (e.g., [36–38]).

Despite their successes, such mixed membership modeling approaches are limited in their ability to resolve fine details of cellular heterogeneity within tumors. They can typically resolve up to about ten distinct cell types, potentially enough to provide representatives of a few major cell clusters but far short of the hundreds of genetically distinct cell populations one can identify in single-cell studies [10, 39, 40]. Furthermore, the difficulty of resolving more than a few cell populations means that each computationally deconvoluted cell type is in reality a noisy average of many genetically similar clones, rather than a single well-defined genetic state. This limitation arises because of the inherent difficulty of resolving mixtures in high dimensions, particularly when distinguishing very similar subpopulations from one another or when distinguishing low-frequency sub-populations from noise.

In the present work, we propose a methodological improvement on genomic unmixing to take advantage of the fact that mixed genomic data from cells evolving according to an evolutionary tree could be expected to have a mathematical substructure unexploited by prior methods. In particular, if one assumes that cell populations across tumors evolve approximately by sampling evolutionary trajectories from a common oncogenetic tree model then we would expect that point clouds produced by representing tumors as points in a genomic space (e.g., by gene expression or gene copy numbers) would yield a finer-scale structure than the uniform simplices assumed by prior work. Instead, they would be expected to yield simplicial complexes: conjunctions of low-dimensional sub-simplices, corresponding to distinct tumor subtypes, joined to one another via lower-dimensional surfaces corresponding to shared ancestral cell populations. Indeed, this kind of subsimplicial structure was noted in earlier studies [16, 23] but has, to our knowledge, not been exploited by any tumor unmixing methods yet developed. By performing mixture analysis in these low-dimensional subspaces rather than the full dimension of the complete point cloud, we hope to avoid the computational challenges and uncertainty introduced by deconvolution in higher dimensions while simultaneously extracting additional information about commonalities within and between tumors useful for reconstructing common cell types and intratumor evolutionary trajectories between tumors.

**Fig. 1:**
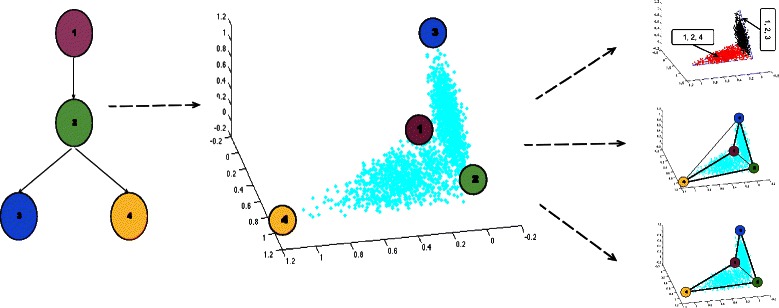
Comparison of the simplicial complex model to other approaches for interpreting tumor genomic data. A simple hypothetical evolutionary tree model (left) describing possible progression pathways of a tumor from an initially healthy cell (1), to an early progression state (2), which then diverges into two subtypes distinguished by two possible late progression states, (3) or (4). If each tumor represents evolution along one of the two subtypes then we would expect plotting many tumors in a low-dimensional representation of gene expression space to yield a point cloud describing mixtures of cell types (1,2,3) or (1,2,4), resulting in a geometric structure consisting of two triangular point clouds joined at an edge (middle). Conventional clustering analysis such as k-medioids with the appropriate distance metric separates this structure into two clusters (top right) that conflate the shared cell states (1,2) and thus provide poor representations of differences in underlying cell populations (3) and (4). Prior approaches to unmixing, such as Tolliver et al., [23] with the correct parameters and under sufficient noise constraints, reconstruct four populations interpreting each tumor as a mixture of all four, introducing high error because the resulting tetrahedral simplex (center right) is poorly populated with data points. The proposed simplicial complex approach here explicitly searches for an overall point cloud that is composed of lower-dimensional subsimplices (bottom right), potentially providing better resolution of mixture compositions in individual tumors and direct ability to infer aspects of the evolutionary process from the geometric structure

Figure 1 shows an overview of the central concept of the work for a toy evolutionary model for two related tumor subtypes. The figure illustrates how an evolutionary scenario might give rise to a structured point cloud consisting of two lower-dimensional subspaces. It then illustrates how the point cloud might be resolved by traditional clustering methods, by prevailing single-mixture deconvolution methods, and by our proposed simplicial complex method.

Below, we present our proposed approach for tumor unmixing via simplicial complexes and validate its effectiveness. We first describe the approach in more detail, presenting an overall pipeline for simplicial complex inference from point clouds of tumor genomic data. The pipeline introduces additional innovations in clustering to identify discrete subspaces of a genomic point cloud and in scoring geometric simplex models for genomic data. We then validate the approach in comparison to our earlier simplex method and an alternative Gaussian mixture model method on a collection of synthetic data. Finally, we demonstrate the method on RNASeq breast cancer (BRCA) data from The Cancer Genome Atlas (TCGA) [35], showing that it is able to identify a simplicial substructure with significant correspondence to known breast cancer subtypes and to build genomic profiles of inferred major cell populations across subtypes.

## Methods

### Model

Computationally, the goal of our overall analysis is to take a data set of genomic measurements on a set of tumors and return a mixed membership model describing inferred genomic measurements of major cell types across tumors and the fractional representation of each cell type in each tumor. We assume input data are given in the form of a set of *g* measurements per tumor across *s* tumors or tumor subsamples, organized into a matrix *M*∈**R** ^*g*×*s*^. Depending on the data set, the *g* measurements may be RNA or DNA copy number values or other probes to genomic state (e.g., overall SNV counts or methylation fractions). For simplicity of exposition, we will refer to the measurements as genes, but they may represent probes at resolutions higher (exon, single base) or lower (genomic region, pathway) than single genes. We will refer to the *s* input samples as tumors, although they may correspond to distinct regional measurements from single tumors. We will assume below that each probe’s measurements have been normalized and recentered to yield a Z score (mean zero and variance one) across samples.

The method attempts to infer a set of mixture components $C = {\vec {c_{1}},\ldots,\vec {c_{k}}}$, where each $\vec {c_{i}}={c_{i,1},\ldots,c_{i,g}}$ is an inferred genomic profile of an unobserved “pure” cell type. Each observed sample $\vec {s_{j}}$ can be approximately explained as a convex combination of components $\vec {s_{j}} = f_{j,1}\vec {c_{1}} +f_{j,2}\vec {c_{2}}, \ldots, +f_{j,k}\vec {c_{k}}+\epsilon _{j}$ for $\sum _{i=1}^{k}\,{f_{j,i}}=1, f_{j,i} \ge 0$. The mixture fraction terms *f*_*j*,*i*_ are collectively denoted by a *g* by *k* matrix *F* and *ε*_*j*_ represents a presumed Gaussian noise in each measurement assumed independent across measurements. The major goal of our computational method, then, is to infer *C* and *F* from *M*, as in prior unmixing approaches.

### Algorithmic overview

**Fig. 2:**
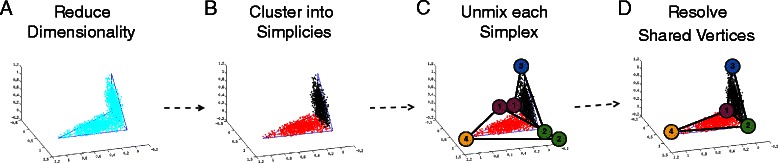
Workflow of the proposed simplicial complex model. We first reduce ambient dimension of the data via PCA to facilitate geometric analysis. We then fit simplices to each of the lineages using a shortest-path weight as an estimate of similarity between 2 points. We then use a novel objective function to robustly unmix each of the lineages. We reconcile shared sub-populations across lineages by training the noise based on several replicates of each of the robust unmixing runs. After a representative model has been derived from the replicates, we abstract the simplicial complex into a phylogeny, based on the dimensionality and connectivity of the simplices in the simplicial complex

While in principle it might be preferable to identify a single objective function for the complete inference process, in practice, the complexity of problem-specific data handling and the disparate classes of algorithms involved make it difficult to effectively pose and solve simplicial complex inference as a single optimization. To produce a practical implementation of the simplicial complex approach, we therefore instead break the computation into a series of discrete steps. The process is illustrated in Fig. 2 and summarized as follows:

(0) Perform dimensionality reduction on *M* to produce a low-dimensional representation *M* ^′^ of dimension *g* ^′^ by *s*, where *g* ^′^≪*g*.

(1) Cluster points in *M* ^′^ to identify subsimplices.

(2) Unmix each cluster to provide a distinct subsimplex and set of vertices per cluster.

(3) Merge nearby vertices across clusters to form a single simplicial complex collectively defining a vertex set *C* ^′^ and mixture components *F*.

(4) Map the components *C* ^′^ from *g* ^′^-dimensional space to *g*-dimensional space to yield a final set of *g*-dimensional mixture components *C*.

We now describe the methods behind each of these steps in detail.

#### Dimensionality reduction (0)

As a preprocessing step, we reduce the initial *g*-dimensional data set to a set of dimension *g* ^′^=*k*−1, where *k* is a user-specified target maximum number of cell types to infer. This step is done to reduce noise in the data and avoid problems of poor scaling in dimension that affect the subsequent computational geometry algorithms. For this purpose, we use principal components analysis (PCA) [41], which projects the initial input *M* into a form *M* ^′^ *V*+*A*, where *M* ^′^ is the reduced representation of *M*, *V* is a set of *k*−1 orthogonal basis vectors of *M*, and *A* is a residual constant translation vector.

Various alternative methods for dimensionality reduction might also be options here, such as independent components analysis (ICA) [42], or any of a variety of non-linear dimensionality reduction methods in the literature [43, 44]. Such methods might lead to better performance depending on the type of data, the degree to which the linear mixture assumption is valid, and the volume and noise-characteristics of the data available. We stick with PCA for the proof-of-concept method developed here, though, because of its simplicity and widespread use in genomic applications.

The number of principal components used involves a trade-off between keeping enough axes of variation to distinguish clonal populations while discarding enough to avoid the poor scaling in dimension of the simplex inference step of the algorithm. We use 10 principal components in the present work because it slightly upper-bounds the number of distinct cell populations we seek to infer.

#### Clustering data into putative subsimplices (1)

The next step in our inference is to identify subgroups of points (tumors) that correspond to distinct low-dimensional subsimplices. This step can be treated as a clustering problem, but differs from standard clustering problems in its objective and requires somewhat specialized methods. In particular, whereas standard clustering typically looks for subsets of points that are separated in space, the present application requires finding subsets of points that are contiguous but occupy distinct subspaces in a larger ambient space. To address this unusual variant of clustering, we use a distance measure based on one originally proposed for the related problem of manifold learning [45]. Specifically, we first establish a complete graph on all samples, with edges weighted by the square of the Euclidean distance between each pair of samples. We then define the distance between any two samples *s*_*i*_ and *s*_*j*_ to be the length of the shortest path between *s*_*i*_ and *s*_*j*_ in the graph. This measure is intended to approximate the geodesic distance, i.e., the distance between two points traveling through the point cloud itself. Intuitively, the clustering method seeks to distinguish points in distinct subsimplices based on the observation that the distance between two points in the same subsimplex should be approximately the same whether distance is measured within the point cloud (geodesic) or via straight-line the distance between the points (Euclidean), while the geodesic distance will generally be longer than the Euclidean distance for points in distinct subsimplices [45, 46]. Given this distance measure, we can then apply a basic clustering algorithm to separate clouds of points in separate subspaces.

We perform clustering via the k-medoids algorithm, an extension of the more standard k-means clustering algorithm [47]. K-means seeks to optimize *k* cluster centers, each inferred to be the mean of a set of points *x* assigned to that cluster, i.e., *k*_*i*_=*m* *e* *a* *n*(*x*∈*K*_*i*_). In k-medoids, by contrast, a cluster center is computed as the data point nearest to the mean of the points assigned to a particular cluster, i.e., $k_{i}=p | p \in data,\underset {p}{\arg \min } ||p-mean(x\in K_{i})||$. This small difference in the method better serves our goal of finding clusters representative of tumor evolutionary data because such a method prevents inference of cluster centers that are not in the subspaces spanned by the data.

#### Unmixing each cluster (2)

Once we have defined distinct clusters in the data, we next unmix data within each cluster via a variant of our prior robust geometric unmixing method [23], which provides a noise-tolerant simplex-fitting assignment by approximately fitting a simplex around a point cloud. The vertices of the simplex will then correspond to inferred mixture components. We modify the prior method in one important respect, however. The prior method used as its objective function the following:

(1) $${} {\fontsize{9.6pt}{9.6pt}\selectfont{\begin{aligned} \underset{K}{\min} \sum{|x_{i}-KF_{i}|_{p}+\gamma \log(vol(K))}; \forall F_{i}: \mathbf{1}({F_{i}^{t}})= 1, F_{i} \ge 0 \end{aligned}}}  $$

where *K* is a matrix of the inferred vertices of the simplex, *F* is the mixture fraction describing amount of each vertex attributed to each input data point, and *vol* is the volume of the simplex. This formula effectively treats the volume of the simplex as a measure of goodness of fit, balanced by a noise term penalizing for data points outside the simplex. In the present work, we instead use the following objective function:

(2) $${} {\fontsize{9.6pt}{9.6pt}\selectfont{\begin{aligned} \underset{K}{\min} \sum{|x_{i}-KF_{i}|_{p}+\gamma \log(mst(K))} \forall F_{i}: \mathbf{1}({F_{i}^{t}}) = 1, F_{i} \ge 0 \end{aligned}}}  $$

where *mst* is the length of the minimum spanning tree on the simplex vertices. This alternative formulation provides a more straightforward interpretation of the problem in terms of an inferred evolution model among cell types, effectively scoring the quality of a simplex by the parsimony score of a tree connecting its vertices. Informally, it says that a “good” simplex is one that yields a parsimonious evolutionary tree by which its vertices could have evolved from a common ancestor. It is also intended to resolve the problem of scores being incompatible between subsimplices of distinct dimension by ensuring that the cost of every subsimplex has the same units. This change makes it possible to sum contributions between subsimplices and define a single quality of fit for the full simplicial complex.

Optimization is performed through the fmincon function of the Matlab optimization toolbox, which utilizes an interior-point method of constrained optimization. The algorithm works by taking as input the data points *x* for a given simplex, and an initial guess of the vertices *K*, determined by the minimum volume enclosing simplex (MVES) algorithm, presented by Chan and others [48]. Then, the computation of the mixture fractions *F* are found by holding *K* and *x* constant, and solving for the first term of the objective function, which is equivalent to a least-squares formulation solvable using the Matlab function lsqlin, given that the *F* are constrained to be nonnegative and sum to 1. In the next iteration of the evaluation of the objective function, the mixture fractions *F* are fixed, and the values for *K* are varied. The iterative process repeats until convergence, or a user-specified number of maximum iterations are exceeded.

In subsequent steps, we also require estimates of uncertainty of the simplex fit. To derive these estimates of uncertainty, we run bootstrap replicates (see, for example, [49]) of the simplex inference procedure for each cluster, using 10 replicates and performing a maximum matching of vertices between replicates for each cluster, in order to identify a mean position and a variance in the position for each vertex of each cluster.

#### Probability model

The objective function defined above is motivated by a probabilistic model combining an assessment of fit of data to the simplicial complex and an assessment of intrinsic plausibility of the complex. For model *θ* and data *x*, we seek to maximize a Bayesian likelihood function

(3) $$ P(x|\theta) P(\theta)  $$

as in our prior work [23]. We effectively assign an exponential prior to the likelihood of the data given the model, yielding exponential decreases in the probability of observing data as it becomes increasingly far from the surfaces of the inferred simplicial complex. That assumption corresponds to the following function:

(4) $$ P(x|\theta) P(\theta) \propto exp\left(-\sum{|x_{i}-KF_{i}|_{p}}\right) mst(K)^{-\gamma}  $$

In practice, it is convenient to work in the negative log domain, seeking to minimize

(5) $$ \sum{|x_{i}-KF_{i}|_{p}}+\gamma \log(mst(K))  $$

The model uses two hyperparameters, *γ* and *p*, for the regularization of the prior on simplicies and the order of the distance measure used to account for noisy simplex fits. We set *γ* in the present work based on prior analysis in Tolliver et al. [23] connecting *γ* to the estimated noise level of the data. Recent estimates of Su et al. [50] put RNAseq noise level in approximately a range of 5 % to 40 %, with the sort of HiSeq data we examine here towards the low end of that range. We tested our method on simulated data of 1,000 data points in 20,000 dimensions reduced to 10 PCs using a range of *γ* values from 1 to 15 (consistent with 1 to 15 % noise) with 10 replicates per parameter value. The resulting root mean square deviations (RMSDs) of reconstruction per model component per dimension were not significantly different across *γ* values, with a low of 24.9 at *γ*=14 and high of 27.8 at *γ*=5. We thus conclude that the method is not highly sensitive to *γ* in this range and chose *γ*=10. *p*=1 was set based on an analysis of data noise from Tolliver et al. [23] suggesting that noise in data in that study was best fit by a log-Laplacian distribution, leading to an L1 norm as the appropriate log likelihood term. This is in contrast to the more conventional use of L2 norm, which would be implied by log-Gaussian noise.

#### Merging subsimplices into a simplicial complex (3)

The previous steps identify a set of subsimplices, each defined by a set of vertices for which we have mean positions and variances. In the final step, we seek to identify instances in which the same vertex is inferred on multiple subsimplices. To accomplish this task, we identify pairs of vertices on distinct simplices that are sufficiently close so as to be putatively an inference of the same data point. We define two vertices to be equivalent if their Euclidean distance from one another is smaller than the sum of their individual standard deviations in distance estimates, as estimated by bootstrapping over simplex inferences in the prior step. We merge all points determined to be equivalent, joining their subsimplices into a single simplicial complex. Formally, we consider two vertices indistinct if they satisfy the following condition, where *V* is the vertex set mean over the replicates, and *σ* is the standard deviation of a vertex as estimated over the replicates:

(6) $$ \begin{aligned} &\exists v_{1} \in V, v_{2} \ne v_{1} \in V, i \in {1,2,\ldots,|v_{1}|}\\& s.t. \sum \left(\frac{||v_{1}-v_{2}||}{\max{i}(\sigma(v_{1,i} \sigma(v_{2,i})))|v_{1}|}\right) \le 1 \end{aligned}  $$

#### Mapping inferred vertices into gene space (4)

To map the inferred vertices back into the gene space, we can project the inferred vertices *K* using the coefficient matrix determined in the PCA (part 0) portion of the algorithm.

#### Complexity analysis

We have also performed a complexity analysis on the code. Because the algorithm presented has a number of parameters influencing run time, and some components of the algorithm, such as k-medioids, are iterative, the complexity expression can become complicated. Nevertheless, we consider a piecewise breakdown of an upper bound to the running time. The first portion of the code uses PCA to reduce the dimensionality of the data. The bottleneck in that step is computation of the singular value decomposition (SVD) [51], which has a complexity of *O*(*m* *n* ^2^) for *m* data points and *n* ambient dimensions (genes markers in our case). The clustering phase of our technique uses k-medioids [52], which has a complexity of *O*(*k* *m*). The simplex fitting applies the method of Tolliver et al. [23] with our modified objective function, an iterative method whose complexity depends on the number of rounds *T* of iteration, as well as the number of clusters *k*, simplex vertices *d*_*p*_, and principal components *P*. The total complexity of this step is $O(Tkn \max _{p}\{2^{d_{p}+1},nP^{2}\})$ for each of *b* bootstrap replicates. The resolution of each of the simplicies into a simplicial complex is performed by learning a Gaussian mixture model on the *b* bootstrap replicates for each of the $\sum _{p}(d_{p}) + k$ vertices [53], contributing a factor of $O(b*(\sum _{p}(d_{p}) +k))$. As a consequence, then, we can upper-bound the running time of the algorithm by $O\left (\max \left (mn^{2},km, bTkn \max _{p}\left \{2^{d_{p}+1},nP^{2}\right \},b*\left (\sum _{p}(d_{p})+k\right)\right)\right)$. In practice, the bottleneck in run time is the simplex inference step, which is likely to be dominated by the cost of the highest-dimension subsimplex. This can, however, be a substantial improvement over the single-simplex approach, which has run-time exponential in the total number of vertices rather than the maximum number in any subsimplex.

### Validation

#### Synthetic data

Evaluation of the effectiveness of the method is complicated by the lack of true heterogeneous tumor data with known ground truth mixture compositions. We therefore used simulated scenarios to quantify effectiveness of the methods for data sets for which the mixture components, mixture fractions, and simplicial structure are known. We considered four tumor evolution scenarios, each involving a model of tumors evolving into two subtypes.

**Fig. 3:**
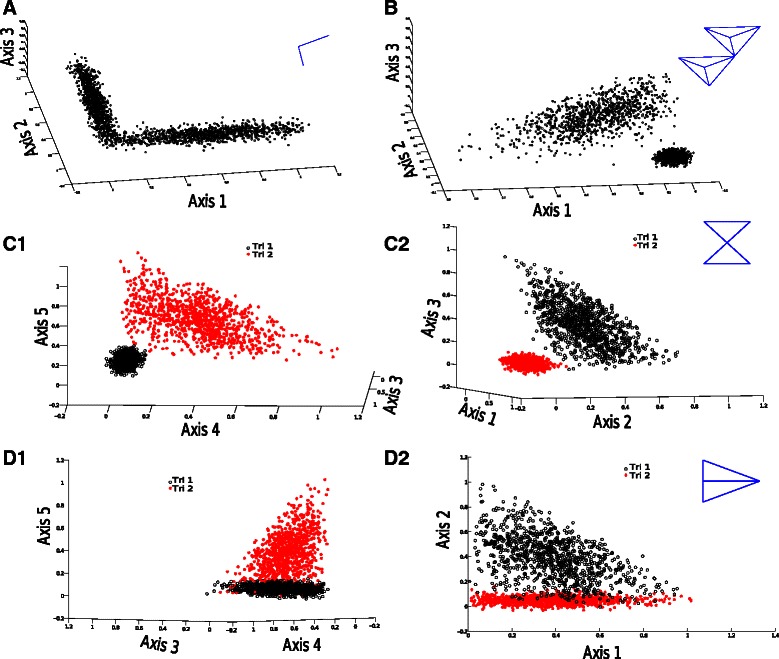
Simulated data sets used for validation. Each data set is visualized as a point cloud plotted in the first three principal components of the gene expression space. Each simulated data set is visualized with noise level 0.1 *σ*, where *σ* is the variance in gene expression level per gene across cell types. Data are displayed in the case of two lines at a point (**a**), two tetrahedra at a point (**b**), two triangles at a point (**c1** and **c2**), and two triangles at an edge (**d1** and **d2**). The triangle cases have been separated to show the connectivity of the non-cosubspatial complexes. Insets in blue provide cartoon illustrations of the projections of cosubspatial versions of the simplicial complexes to facilitate visualization of the true structure of high-dimensional point sets

The scenarios are illustrated in Fig. 3. In scenario A (Fig. 3(a)), we assumed an ancestral healthy cell subpopulation that diverges into two discrete subtypes each with a single progression state. The result is a simplicial complex structure consisting of two lines joined at a point. In scenario B (Fig. 3(b)), we assumed a more complex two-subtype scenario, in which each subtype is defined by early, intermediate, and late progression stages. The resulting simplicial complex structure is a pair of tetrathedra joined at a point. Scenario C (Fig. 3(c)) consists of two subtypes each with its own early and a late progression stages, yielding a simplicial structure of two triangles joined at a point. Finally, scenario D (Fig. 3(d)) consists of a model in which a healthy state evolves into an early precancerous state that subsequently branches into two subtypes of late state. This model results in a simplicial structure of two triangles sharing an edge. For each scenario, we constructed variants of the data set at distinct noise levels from zero to 1 in increments of 0.1*σ*, where *σ* is the standard deviation per dimension in the input data. In each case, the data were simulated with an ambient dimension (equivalent to the number of genes probed) of 25,000. This ensures the comparison is similar to the real data, which contains 20,531 probes across the genome.

For each scenario and noise level, we seek to measure the relative performance of the method at reconstructing the vertices, which correspond to the genetic signatures of the major cell subpopulations, and identifying the mixture fraction of each of the samples with respect to these cell subpopulations. The errors in the mixture fractions are computed as the RMSD per point per ambient dimension. The errors in the vertices are computed per vertex found per ambient dimension.

To provide a basis for comparison, we repeated our analysis using our earlier pure simplex method [16] and an implementation of a Gaussian mixture model method, because Gaussian mixture models are used in some prominent alternative approaches to this problem (e.g., PyClone [28]). It should be noted that PyClone contains significantly more complex machinery than only a Gaussian mixture model, but in order to perform a direct comparison of classical techniques to our method, we have chosen the Gaussian mixture model because it has appeared as a part of the PyClone architecture. We fit Gaussian mixture models using the built-in expectation maximization (EM) approach supplied by Matlab. To invoke the model, we fit the ground truth number of vertices using the data through the command gmdistribution.fit(X,k) where *X* is the expression matrix and *k* was supplied with the correct number of vertices for each simulated scenario (e.g., *k* =7 for two tetrahedra joined at a point). We use a basic implementation of generic Gaussian mixture models because our proof-of-concept method here is designed to work with matrices of pre-aligned copy numbers rather than raw sequence data and our test data is thus incompatible with the widely used software platforms for this problem, which generally work directly on genome-mapped sequence reads.

#### Real tumor data

To demonstrate the applicability of the method to real tumor data, we applied it to RNAseq expression data from The Cancer Genome Atlas (TCGA) [35]. We retrieved all breast cancer (BRCA) RNAseq samples from the RNASeqv2 section of the TCGA data portal (http://cancergenome.nih.gov/), selecting normalized data. The data matrix consists of 1100 tumors profiled at 20,531 expression levels. We then preprocessed data to convert expression values for each gene to Z scores by subtracting the mean expression level of each gene from all observed values for that gene and then dividing the zero-centered expression levels by the standard devation across values for the gene. We then applied our analysis pipeline to the resulting data matrix. We have chosen the RNASeq data due to the availability of a relatively large number of samples compared to other data types. Clinical labels for the tumor types were extracted from the clinically available data in TCGA, with a label of unavailable if one or more of the clinical labels was listed as not available, indeterminate, or not evaluated.

Direct comparison to other published tools for this problem is challenging due to differences in assumptions about inputs and outputs. Our technique is designed to be a generic with respect to input data type, with the only requirement being that the data can be represented as a matrix of samples by genes. Further, our method does not require that the samples be evaluated on a per-allele basis, or that the data be provided as paired normal/tumor samples. This flexibility, however, creates a challenge in comparing and contrasting our work with previous models of tumor purity and tumor evolution. Several of the previous models require significantly more input data, often in the form of paired normal/tumor samples [32]. Other techniques performing similar tasks are suited to using only specific data types, such as specifically-formatted single nucleotide polymorphism (SNP) or array comparative genome hybridization (aCGH) arrays [54]. Other published methods require additional input parameters, such as the relative prevalences of subclones [29] or LOH information [27]. Many of the recent methods are specific to sequence data, rather than the processed expression matrix format we use as input [25, 26, 28, 29]. Still other popular methods in this class solve only the purity estimation problem rather than the problem of finer reconstruction of subclonal architecture solved by our method [24]. These incompatibilities in inputs or outputs make a direct head-to-head comparison of our method with any other available method known to us infeasible. We therefore provide a final validation based on comparison of results of our novel method with published subclone and purity estimation results on TCGA breast cancer samples on which our method has also been run, despite some differences in the specific data used by our method versus the comparative method used to derive the TCGA subclone and purity estimates [31].

## Results and discussion

**Fig. 4:**
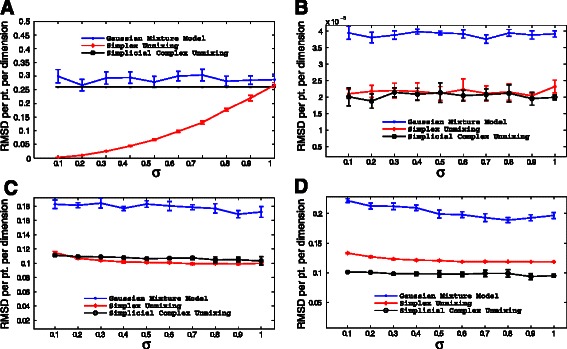
Comparative accuracy of inferring mixture fractions (tumor composition) from simulated data as a function of data noise level. Accuracy is quantified by the RMSD per point per dimension, shown in the example cases of two lines at a point (**a**), two tetrahedra at a point (**b**), two triangles at a point (**c**), and two triangles at an edge (**d**). The inferences are computed as the mean over 10 replicates. The figure shows relative inference accuracies for the simplicial complex method and the comparative simplex and Gaussian mixture model methods

***Validation on synthetic data*** We first examined the ability of the methods to correctly infer mixture fractions across scenarios. Results are shown in Fig. 4. For the simplest scenario, the two-line scenario 1 (Fig. 4(a)), the basic simplex unmixing showed the best performance, with nearly perfect fitting at low noise, although generally poor scaling with noise level. The simplicial complex showed poorer results except at the highest noise level, but high consistency across noise level and independent trials. The primary advantage of the simplicial complex over the simplex is in mitigating the poor scaling of the pure simplex method with dimension of the data by performing geometric inferences in low-dimensional subspaces. It is to be expected that this advantage would be minimal for problem instances with small total dimension. The Gaussian mixture model did slightly worse than the simplicial complex on this scenario and showed higher variance run-to-run, but similarly good scaling with noise level. For the remaining scenarios, results were qualitatively similar in each case. The simplicial complex method yielded slightly better accuracy than the simplex method in scenario 2 (Fig. 4(b)) and scenario 4 (Fig. 4(d)) and slightly worse accuracy in scenario 3 (Fig. 4(c)). Both geometric methods substantially outperformed the Gaussian method for scenarios 2–4. Aside from the anomolous results for the simplex method in scenario 1, all of the methods show minimal sensitivity to noise in determining mixture factions.

**Fig. 5:**
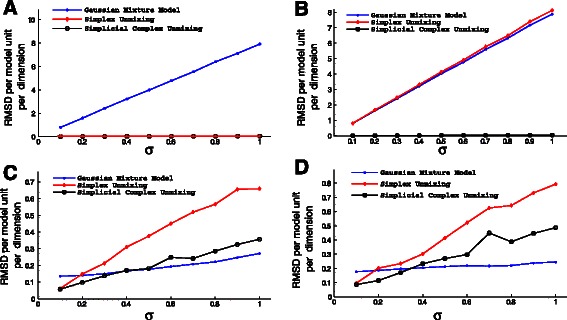
Comparative accuracy of inferring subpopulation genomic signatures (cell types) from simulated data as a function of data noise level. Accuracy is quantified by the RMSD per model component per dimension, shown in the example cases of two lines at a point (**a**), two tetrahedra at a point (**b**), two triangles at a point (**c**), and two triangles at an edge (**d**). The inferences are computed as the mean over 10 replicates. The 10 replicates are used to form the merged consensus simplicial complex model. The figure shows relative inference accuracies for the simplicial complex method and the comparative simplex and Gaussian mixture model methods

We next examined ability of the methods to reconstruct the correct vertices, representing inferences of the expression profiles of the unmixed states. The results for each scenario are shown in Fig. 5. For scenario 1 (Fig. 5(a)), both geometric models led to substantially more accurate inferences than the Gaussian mixture models. Scenario 2 (Fig. 5(b)), which involved the highest total dimension, showed similar results for the simplicial complex and Gaussian methods. The simplicial complex again yielded substantially better accuracy and scaling with error than Gaussian. In this case, however, the simplex method closely tracked the Gaussian. Scenarios 3 (Fig. 5(c)) and 4 (Fig. 5(d)), which have intermediate total dimension, show qualitiatively similar outcomes to one another. The simplicial complex method shows the lowest error at low noise levels, but the Gaussian mixture model the lowest error at high noise levels. The pure simplicial method performs worst in every case except zero noise.

These scenarios allow us to draw a few general conclusions about the advantages of the simplicial complex approach. The geometry-based methods have a significant advantage with respect to mixture fraction inference, presumably because they are based on an explicit model of the expected point cloud structure of linear mixtures. The advantage may be overstated here, though, since the data is simulated directly from the model assumed by these methods, while real data might be expected to deviate more significantly from that model. The advantage of simplicial complex inference would be expected to be most pronounced when there is a large gap between the intrinsic dimension of the full data set and that of its low-dimensional subspaces. From a biological perspective, this condition is most likely to be met when distinct tumors subsample from distinct subsets of a set of fundamental cell types, as might occur if tumors partition early into distinct subtypes each of which has its own defined progression stages. This expectation is generally consistent with the better performance of the simplex method on scenario 1 and simplicial complex on scenario 4, with both showing essentially the same accuracy on scenarios 2 and 3. The portrait is more complex with respect to vertex inference. Both geometric methods outperform the Gaussian method for very low-dimensional data and the simplicial complex method outperforms both others with high-dimensional data. For intermediate dimensions, the Gaussian method shows significantly better noise tolerance, leading to a preference for the simplicial complex method when working on low-noise data and the Gaussian mixture model when analyzing high-noise data.

Prior work of Su et al. [50] examining noise levels of various RNASeq platforms estimated noise ranges from approximately 5 % to approximately 40 %, dependent upon the platform [50]. We would thus expect real modern RNA-seq data sets to fall in the low-to-middle region of noise ranges examined in the synthetic scenarios, where the superior tolerance of the Gaussian method to high noise is not yet an appreciable advantage.

Although run time is not expected to be a particular strength of the newly proposed method, we do compare run times of the simplicial complex method, Gaussian mixture model, and simplex unmixing method on one run of 400 data points for each of the four scenarios to evaluate practicality of our new method on realistic sizes of data set. For the scenario of two lines joined at point, run times were 173 s for the simplicial complex method, 0.794 s for the simplex method, and 0.0955 s for the Gaussian mixture model. For the scenario of two tetrahedra joined at a point, run times were 297 s, 4.62 s, and 0.112 s for the simplicial complex, simplex, and Gaussian mixture model respectively. For the scenario of two triangles joined at a point, run times were 829 s, 1.22 s, and 0.133 s for the simplicial complex, simplex, and Gaussian mixture model, respectively. For two triangles joined at an edge, run times were 554 s, 5.94 s, and 0.147 s. These results suggest that the greater complexity of the simplicial complex approach does come at a significant cost in run time.

However, we believe these run times should be a reasonable tradeoff for better accuracy for typical users, considering this analysis would generally only need to run once per replicate.

**Fig. 6:**
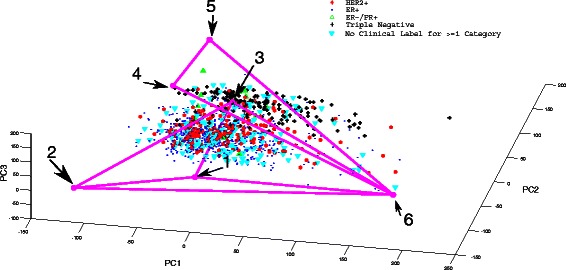
Simplicial complex reconstruction on real RNAseq data from TCGA breast cancer samples. Data is plotted in a two-dimensional projection derived from the first three principle components, manually rotated to best display separation amoung discrete subregions of the tumor point cloud. Inferred vertices are shown as magenta points, labeled with integers corresponding to the labels in Table 1. The simplicial complex is shown by magenta lines. The underlying point cloud is shown with four point types corresponding to an inferred labeling by clinical subtypes: HER2+ (red asterisks), ER+ (blue points), ER-/PR+ (green triangles), triple-negative (black crosses)

***Application to real tumor data*** We applied our clustering method in order to partition data points into simplices, selecting two clusters empirically. We based the selection on the relatively low variance in cluster size for the choice of two clusters when compared to the variance in cluster size of larger numbers of clusters over multiple clustering replciates. After clustering, we estimated seven total vertices in the resulting simplicial complex, comprising two simplices of four vertices each, using a prior heuristic test of the eigenvalue spectrum [23]. Two of the vertices of one tetrahedron were sufficiently similar to one another, as determined by the criterion of Eq. (3), that they were merged to form one vertex. The final resulting simplicial complex structure then consisted of a triangle and a tetrahedron joined together at a point, yielding a total of six subpopulations. The resulting simplicial complex and associated point cloud appear in Fig. 6, with inferred vertices labeled 1–6. The tetrahedral subsimplex is defined by vertices 1, 2, 3, and 6 and the triangular subsimplex by vertices 4, 5, and 6.

**Table 1:** Summarized terms for genes with high Z scores in inferred subpopulations

	Z score ≥3
Subpop. 1	Defense Response, Inflammatory Response, Response to Wounding
Subpop. 2	Myofibril,Contractile Fiber Part, Sarcomere, I band, Actin Cytoskeleton, Z disc
Subpop. 3	*∅*
Subpop. 4	RNA recognition motif and associated terms, RNKP-1, alpha-beta plait
Subpop. 5	Cell cycle and associated terms, M phase, organelle fission, nuclear division
Subpop. 6	ribosome and associated terms, translational elongation, cytosolic part

As a test of whether the results yielded any significant subtyping of tumors, we labeled each observed tumor with one of four labels corresponding to known breast cancer subtypes. We extracted clinicial labels where present from TCGA, and labeled the points as no label available if at least one of the clinical labels was missing. We used immunohistochemistry (IHC) labels if available, and if these labels were not available, we attempted to apply a label obtained by fluorescence in-situ hybridization (FISH). We considered clinicial labels of HER2+, ER+, ER+/PR-, Triple negative, and a tumor with missing clinical labels. To assess whether the inferred structure showed corresponsence with the known subtyping, we applied a chi-squared test for independence to the contingency table comprised of the two simplicies and five labels, yielding a strongly significant chi-squared score of 542.5002 (p-value <0.001). Manual inspection of the simplex fits shows that the triangular simplex is strongly associated with triple-negative tumors and the tetrahedral simplex with the remaining classes. Within the tetrahedral simplex, HER2+ tumors are shifted towards vertex 3 and ER+ tumors towards vertices 1 and 2. Insufficient numbers of ER-/PR+ tumors are observed to discern any bias in their placement.

We similarly examined the distribution of PAM50 [55] subtyping labels (Basal, HER2+, Luminal A, Luminal B, and Normal) relative to our simplex asignments. We assigned these labels by identifying the nearest PAM50 expression signature from Parker et al. to each sample in Euclidean distance. Where assignment of gene names between TCGA samples and PAM50 genes was ambiguous, we used the first suggested gene name from the BioDB gene ID Converter System [56]. We then constructed a contingency table comparing our two simplex labels against the five PAM50 labels. We found a strongly significant chi-squared score of 775.2952 (p-value < 0.001). We further performed a finer comparison of PAM50 labels to our mixture components by establishing a contingency table of PAM50 labels versus most prevalent mixture component for each tumor. We found a strongly significant chi-squared score of 1128.6668 (p-value <0.001). These results suggest that our method’s inferred simplicial structure is strongly correlated with accepted breast cancer subtypes.

We further tested for potential functional significance of the six inferred subclonal populations identified by vertices of the simplices. We identified potential markers of subclonal populations by selecting genes in each of the inferred populations with Z score less than −3 or greater than 3. In order to link the differentially expressed genes with cancer types, we used DAVID [57] to identify significantly over- or under-represented functional categories. The functional annotation cluster results are presented in Additional file 1, while a summary of terms is presented in Tables 1 and 2. We note that some terms are nearly repeated due to DAVID’s use of multiple ontology resources.

**Table 2:** Summarized terms for genes with low Z scores in inferred subpopulations

	Z score ≤−3
Subpop. 1	*∅*
Subpop. 2	Chemokine and associated terms, Cytokine and associated terms, interleukin-8-like
Subpop. 3	Alternative Splicing, Splice Variant, phosphoprotein, polymorphism, sequence variant
Subpop. 4	Signal, Signal peptide, glycoprotein, glycosylation site: N-linked, disulfide bond
Subpop. 5	Icosanoid, unsaturated fatty acid, alkene, leukotriene, transmembrane protein, lipid
Subpop. 6	Zinc and associated terms, c2h2-type

The DAVID analysis showed the upregulated genes of vertex 1 to be enriched for genes related to inflammation response. The association of inflammation-related expression with tumors is supported by extensive literature. For reviews of the role of inflammation in cancers, see, for example, [58–61]. We speculate this vertex may reflect a confounding influence of immune cell infiltration in the tumors. The upregulated genes of vertex two disproportionately involve contractile fiber and actin filament-related expression, which is likewise an unsurprising result given known relationship of these functions to cancers [62, 63] and their prior use as a marker for predicting clinical outcomes of carcinomas [64]. Vertex 2 shows downregulation of genes related to cytokines and taxis, processes whose dysregulation is linked to the development of breast cancers in general [65–67]. Elevated cytokine expression has been linked specifically to ER- breast cancers [68], which seems consistent with the observation that this vertex is most closely associated with an ER+ tumor subpopulation. Vertex 3 shows elevation for genes associated with splice variation, sequence variation, and polymorphism, suggestive of hypermutability processes associated generically with breast cancers [69–73]. Vertex 4 shows upregulated genes related to RNA binding motifs, which have also been linked to pathways associated with breast cancer [74–76]. Vertex 4 also shows underexpression of genes associated with glycosylation, which has also previously been linked to breast cancers [77–79]. Vertex 5 shows increased expression for genes associated with the cell cycle and mitosis, a generic feature of many cancers [80]. Different mechanisms of upregulating the cell cycle have, however, been linked with distinct clinical subtypes of breast cancer [81]. Similarly, vertex 5 shows decreased expression for genes associated with metabolic processes, another broad category generically linked with development of a wide variety of cancers [82, 83]. Vertex 6, which joins the two subsimplices, is most closely associated with ribosomal genes and depleted for genes related to zinc metabolism. Based on location of this vertex at the junction of the two subsimplices, we speculate it corresponds to healthy stromal cells infiltrating all tumor types. A relatively high expression level of ribosomal genes, a generic housekeeping marker, would be consistent with that interpretation. There is also evidence for generic upregulation of zinc metabolism in breast tumors [84], which would be consistent with a normal cell subpopulation showing comparatively reduced expression of zinc-related metabolism.

**Fig. 7:**
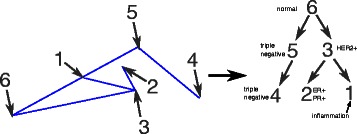
Minimum spanning tree of simplicial complex derived from the TCGA data and a model of the data as a phylogeny. We compute the minimum spanning tree of the simplicial complex found during unmixing of the RNASeq data to learn the phylogeny of subpopulations inferred by the simplicial complex unmixing. Labels for the vertices carry over from the previous figure and Table 1. We use the minimum spanning tree to define tree structure among the vertices. We label each vertex manually based on examination of the simplicial complex fit and the DAVID categories associated with the vertices. We note that although population 1 is included where it is assigned in the tree, we speculate that it reflects infiltration of immune cells not phylogenetically related to the tumor cell populations

In Fig. 7, we attempt to synthesize the minimum spanning tree found during simplex fitting with visual examination of the correspondence between the simplicial complex and the point cloud into an intepretation of the tumor progression process. At a high level, the simplicial complex shows a broad separation between the triangular subsimplex and the tetrathedral subsimplex. The most plausible interpretation would be that the triangular subsimplex corresponds to the basal-like tumors. The tetrahedral subsimplex shows discrete regions corresponding to HER2+, ER+, and ER-/PR+ tumors, with vertex 3 corresponding to HER2+ progression and vertices 1 and 2 to ER/PR progression. We attributed vertex 1 specifically to immune cell infiltration based on the DAVID functional analysis, and we therefore believe this vertex is a confounding factor in evolutionary tree inference since it shows up as part of the mixture fraction in many tumors but is presumably not on a progression pathway with the other cell populations. We therefore believe that a more correct model would be consist of progression from normal cells (6) through two independent triple-negative steps (4,5) or alternatively progression from normal to either a HER2+ (3) or an ER/PR (2) pathway. The seeming mixture of HER2+ and ER+/PR+ cells could then be explained by the confounding effects of these populations both exhibiting mixture with normal and immune cell populations in violation of the assumptions of the phylogeny.

Total run time on the real data for our method was 242 s. By comparison, the simplex method required 13.5 s on this data set and the Gaussian mixture model 0.271 s.

***Comparison with prior methods*** We can provide some comparison of our method to alternative approaches based on similarity of our results with those reported in Oesper et al. [31] in benchmarking their method (Theta2) against the widely used purity analysis tool ABSOLUTE [54]. They examined two TCGA breast tumor samples for which we have also derived results using the TCGA RNAseq data. Oesper and other achieved an estimate of three total populations in each of samples TCGA-A2-A0EU and TCGA-AO-A0JL [31]. Our method also found three total populations for these samples. ABSOLUTE [54] does not determine a total number of subpopulations since its goal is purity analysis, so we cannot directly compare against ABSOLUTE in that sense; however, our method found a normal subpopulation proportion in TCGA-AO-A0JL-01A-11R-A056-07 of 0.601 (purity 0.399), while ABSOLUTE yielded 0.500 purity in Oesper et al.’s analysis. For the same sample, Theta2 estimated subclones in proportions of 0.57 and 0.3, while our method yielded estimates of 0.34 and 0.05. In a second sample, TCGA-A2-A0EU-01A-22R-A056-07, our method estimated a total tumor purity (non-normal fraction) of 0.6359, compared to ABSOLUTE’s value of 0.49. Theta2 yielded subclone estimates of 0.427, 0.346, and 0.227, while our estimates were 0.5434, 0.3641, and 0.0925. We also note that the authors of ABSOLUTE validated their method on a proprietary breast cancer dataset unavailable for further analysis, finding mean purity 0.67 and standard deviation 0.07 across this population [54]. We cannot compare to their results on the same data, but treating our non-normal frequency as a purity estimate gives mean 0.614 and standard deviation 0.156 for the TCGA breast cancer samples. These results are only anecdotal and provide no way of comparing relative accuracy of the three methods, but are suggestive that our method yields at least qualitatively similar purities and clonal compositions to these methods.

A more direct comparison of comparable predictions on a common set of tumor samples was possible with ESTIMATE [85], a tumor purity tool that had previously been applied to 480 TCGA breast tumor samples, representing a subset of the data unmixed here, in the process of analyzing a larger pan-cancer data set. ESTIMATE separately derives scores intended to measure degrees of stromal and immune infilitration, which are then integrated with other analysis to derive an overall purity estimate. (Additional file 2: Figure S1) provides dot plots comparing ESTIMATE scores with component mixture fractions from our method. (Additional file 2: Figure S1 (A))) plots ESTIMATE immune scores against our component 1 fraction, which we attributed to immune infiltration. While our method assigned component 1 only to a subset of samples, it did nonetheless show a strongly significant Spearman correlation coefficient of 0.264 (*p*< 0.00001). (Additional file 2: Figure S1 (B))) plots ESTIMATE stromal scores against our component 6, which we attributed to normal stromal cell contamination. These two scores showed an insignificant correlation of 0.0271, however. Finally, (Additional file 2: Figure S1 (C))) plots ESTIMATE percent purity inferences against the sum of our components 2–5 mixture fractions, which we attribute to the non-stromal, non-immune cell populations and hence to tumor purity. Our purity estimates tend to occupy a narrower range of values than the ESTIMATE ones, but the results show significant Spearman correlation coefficient of 0.180 (*p*< 0.00001). These results cannot be considered a true head-to-head comparison of the two methods, since they are designed for different purposes and work on different data, and because we lack a known ground truth to which to compare. Nonetheless, they do provide some indication that our method can infer mixture components consistent with separating tumor and non-tumor cell populations. The lack of correlation between our identified normal component and the ESTIMATE stromal score might reflect a misidentification of our component 6 as stromal, or simply an incomparability of the two scoring schemes.

## Conclusions

We have developed a novel method for deconvoluting mixtures of genomic data with the goal of more accurately reconstructing cellular progression processes from bulk tumor genomic data. The method is designed to take better advantage of an underlying geometric structure one would expect from data produced by lineages sampled from a common evolutionary tree. Specifically, the work advances prior geometry-based unmixing work, which uses a representation of mixed membership modeling in terms of fitting simplices to high-dimensional data, by instead seeking fits that correspond to unions of disjoint subsimplices of lower dimension. This approach is intended to improve handling of non-uniform mixtures and minimize problems of poor scaling in intrinsic dimension that plague all standard methods for blind-source unmixing. Implementing this approach required additional innovation in clustering to handle the special problem of identifying clusters that are spatially contiguous but occupy distinct subspaces. It also introduced a novel objective function for geometric fitting intended to better reflect the interpretation of tumor mixed membership models as products of an evolutionary process. Tests on simulated data demonstrate that the method provides markedly improved ability to correctly identify fractions of cells in distinct subsamples relative to the common alternative of Gaussian mixture modeling while improving on the basic simplex approach for data sets with a significant gap between the dimension of the full data set and its subspaces. At the same time, it substantially improves inferences of vertices over the pure simplex method, although with poorer handling of noisy data than Gaussian methods. We attribute this improved accuracy to its ability to explicitly model the fact that distinct mixtures will tend to share only defined subsets of cell types, removing the confounding influence of cell types completely absent from particular tumors.

The current method demonstrates the power of more sophisticated geometric models, but also leaves many avenues for future improvement. One current weakness of the approach is the difficulty of accurately estimating the dimensionality of subsimplices, a challenging problem for sparse, noisy data sets embedded in high-dimensional spaces. This problem might be better solved with approaches specifically designed for the data size and noise characteristics of genomic data, which are quite different from the much larger numbers of data points and smaller ambient dimensions frequently assumed by the dimensionality estimation literature [86]. There is likewise room for improvement in the clustering method, which tends to yield significant error at boundaries between sub-simplices and might benefit from the introduction of soft clustering to better model this uncertainty. One consequence of our simplicial complex model is that a sample cannot be inferred to have any contribution from a component outside its simplex. This feature may lead to incorrect inferences of mixture assignments if some cell populations violate the assumption of progression along a phylogeny, as can occur when accounting for the influence of non-tumor cells within a tumor sample. This potential problem becomes apparent in our interpretation of our inferred component 1 for the real tumor data as representing immune cell infiltration. By assigning that component to a subsimplex, our method then concludes that tumors not assigned to that subsimplex have zero immune component. The method might be improved, at least for application to gene expression data, by allowing for components that do not arise from a common tumor phylogeny and thus may not obey the assumed simplicial complex structure. More generally, we expect that the method could be improved by handling the complete process as a optimization over a unified objective function that allows tradeoffs between solution qualities of the discrete steps of our pipeline. In principle, the MST measure we have developed for scoring simplex fitting will extend easily to a single score for the entire simplicial complex. Properly integrating a phylogenetic likelihood with contributions to reflect the quality of the clustering, data fitting, and model regularization could present substantial algorithmic challenges, however. The relatively poor noise tolerance of our method compared to Gaussian mixture models suggests that a hybrid of the two approaches might outperform both.

We further expect that the problem this method is intended to solve — better fitting mixed membership models to data derived from tree-like progression processes — is likely to apply to many other kinds of genomic data. In particular, DNA variation data (copy number or SNV) would be expected to conform much better to linear mixture assumptions and avoid some confounding effects, such as that of immune cell inflitration, and thus provide more reliable inferences of clonal structure of tumors and their evolution. Quantitation accuracy of DNA-seq has not been studied as intensively as for RNA-seq and can in any case be expected to be sensitive to platform and coverage. Nonetheless, a comparative analysis of copy number detection accuracies [87] found that standard DNA-seq could detect CNVs with perfect sensitivity at a 10 % false positive rate, rates suggestive of quantitation accuracy comparable to that of RNA-seq. Studies of synthetic DNA-seq data, such as that created for various DREAM challenges [35, 88, 89], may prove useful in better evaluating the relative success of the proposed methods for DNA-seq compared to RNA-seq and more precisely measuring accuracy of the proposed methods on sequence data with known ground truth.

Finally, we note that the work here is not intended to establish a stand-alone software utility for broad use by experimentalists. Rather, it is intended to provide a proof-of-concept demonstration of a methodological improvement that we believe could benefit many tools for deconvolution of genomic data for cancer research and related fields.

## Availability of supporting data

Supporting materials for this work, in the form of software used for the described analysis, is provided as Additional file 3.

## Additional files

Additional file 1
**Full results of clustered ontology terms from DAVID, first cluster.** These results are for the first cluster using the default parameters of DAVID [57]. The genes were separated based on the intepretation of Z score ≥3 as overexpressed and Z score ≤−3 as underexpressed. Results are shown for each of the six inferred mixture vertices (cell subpopulations). (XLSX 5.44 KB)

Additional file 2
**Comparison of our method with ESTIMATE [** 85 **].** This file provides dot plots comparing mixture fractions from the simplicial complex unmixing method to scores from the ESTIMATE tumor purity estimation program for 480 TCGA breast cancer tumors, presented as Supplementary Fig. S1. We compare results on TCGA breast tumor samples from ESTIMATE to putatively comparable mixture fraction estimates from our simplicial complex method. (**A**) Component 1 mixture fraction, which we attribute to immune contamination, versus the immune score from ESTIMATE. (**B**) Component 6 mixture fraction, which we attribute to normal cell contamination, versus the stromal score from ESTIMATE. (**C**) Sum of component 2–5 mixture fractions, which we attribute to tumor cells, versus ESTIMATE-inferred tumor purity. (PDF 90.3 KB)

Additional file 3
**SCUnmix software package.** This file is a zip archive containing source code used to perform the analysis in the paper, in the form of Matlab “.m” files. Instructions for use are contained in the file README.txt. (ZIP 19.2 KB)

## Abbreviations

FISH: Fluourescence in situ hybridization
CNV: Copy number variant
SNV: Single nucleotide variant
aCGH: Array comparative genome hybridization
BRCA: Breast cancer
DAVID: Database for annotation, visualization and integrated discovery
MST: Minimum spanning tree
LOH: Loss of heterozygosity
SNP: Single nucleotide polymorphism
SVD: Singular value decomposition
