# Supplementary figures and images for: A simplicial complex-based approach to unmixing tumor progression data
Source: BMC Bioinformatics. 2015 Aug 12;16:254. doi: 10.1186/s12859-015-0694-x (PMC4534068; doi:10.1186/s12859-015-0694-x)

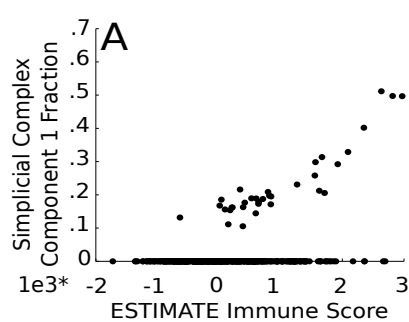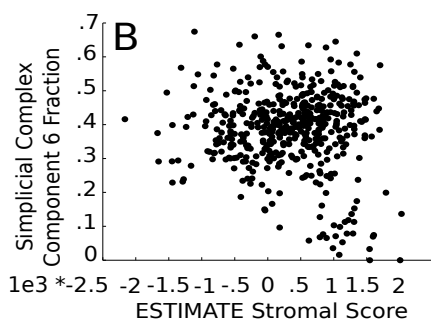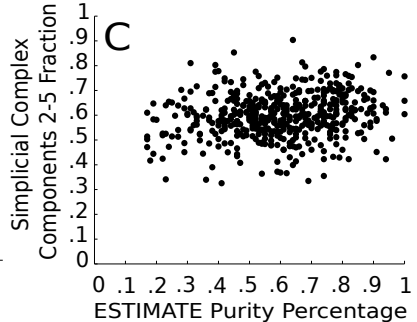

Supplement: Additional file 2 — Comparison of our method with ESTIMATE [ 85 ]. This file provides dot plots comparing mixture fractions from the simplicial complex unmixing method to scores from the ESTIMATE tumor purity estimation program for 480 TCGA breast cancer tumors, presented as Supplementary Fig. S1. We compare results on TCGA breast tumor samples from ESTIMATE to putatively comparable mixture fraction estimates from our simplicial complex method. (A) Component 1 mixture fraction, which we attribute to immune contamination, versus the immune score from ESTIMATE. (B) Component 6 mixture fraction, which we attribute to normal cell contamination, versus the stromal score from ESTIMATE. (C) Sum of component 2–5 mixture fractions, which we attribute to tumor cells, versus ESTIMATE-inferred tumor purity. (PDF 90.3 KB) [file 12859_2015_694_MOESM2_ESM.pdf]
